# Is Social Presence Indeed Present in Remote Social Interactions? A Call for Incorporating Physiological Measures of Synchrony When Assessing the Social Nature of Interpersonal Interactions *via* Videoconferencing Platforms

**DOI:** 10.3389/fpsyg.2022.822535

**Published:** 2022-03-22

**Authors:** Jenny Gutman, Ilanit Gordon, Noa Vilchinsky

**Affiliations:** Department of Psychology, Bar-Ilan University, Ramat Gan, Israel

**Keywords:** social presence, videoconference, physiological measures, dyadic, remote technical communication, telemedicine, biobehavioral synchrony, interpersonal social relations

The recent global COVID-19 pandemic has brought digital communication platforms into the homes of millions of people who never considered using them before. The reduced opportunity to spend time together in person has enabled even those who are less technologically savvy to turn to their computer screens to remain socially connected or active in their workplace (Nguyen et al., 2020). Admittedly, the pandemic has accelerated a process that has been slowly unfolding for a couple of decades: the infiltration of digital remote communication technologies into our everyday social communication routines (Rainie and Wellman, 2012).

In this article we focus specifically on video call and video conferencing internet-based technologies defined as any internet-based communication platform that allows for the reception and transmission of audio-video signals by users in different locations in real time (e.g., Zoom, FaceTime, Skype, Google Meet, etc.) (McGraw-Hill, 2022). This mode of communication has many benefits, expanding our capacity for staying socially connected and exchanging emotional and material support (Nguyen et al., 2021). It allows workers to work remotely, students to continue studying and gain access to education all over the world, and friends and family members to maintain intimate relationships from a distance. The field of telemedicine has been especially influenced by these changes, as many healthcare institutions have increased their use of telecommunication throughout their responses to the pandemic (Bashshur et al., 2020). The trend of increasing demand for teleconferencing and remote collaboration technologies is likely to continue at a fast pace even after the pandemic begins to wane, as individuals and organizations are coming to rely on them as substitutes for face-to-face interaction far more than ever before (Hacker et al., 2020).

Consequently, the need for empirical-based scientific methods for the evaluation of how “social presence technologies” perform is increasingly important. Although these technologies vary, they share a common goal: most were designed, engineered, and manufactured to improve social presence (Biocca and Harms, 2002). Social presence was originally defined as the sense that another person is “real” and “there” when using a remote communication medium (Short et al., 1976). Later definitions included the following: the sense of “being with others” (Heeter, 1992), “the moment-by-moment awareness of the co-presence of another sentient being accompanied by a sense of engagement with the other” (Biocca et al., 2001), “the degree of salience of the other person in the interaction” (Short et al., 1976), and the “feeling that one has some level of access or insight into the other's intentional, cognitive, or affective states” (Biocca et al., 2001).

Whereas, the available research literature has effectively documented many of the psychological, neurological, and behavioral parameters of social interactions in the real world (during which physical presence is a given), the fast expansion of these new digitally mediated communication technologies has outpaced the available research (Meier and Reinecke, 2021). In fact, although the growth of social presence technologies has been accompanied by claims of improved social communication, collaboration, social presence, and performance, there is very little scientific evidence to substantiate these claims (Basch et al., 2020; Correia et al., 2020). We therefore wish to stress the need for empirically driven ways of evaluating the quality of communication and social interaction that these various platforms allow for. We further wish to suggest the adoption of an interpersonal biobehavioral lens as a fitting scientific perspective for bridging the lacunas in knowledge in the existing attempts to conceptualize and measure social presence.

Presently, there are too many definitions and measures for the concept of social presence, precluding the development of a coherent research field (Kreijns et al., 2021). Although it seems that most definitions can be placed somewhere on a continuum between the realness of the mediated situation and the feeling of connection between the participants (Lowenthal and Snelson, 2017), some definitions cannot in fact be classified as lying anywhere on this continuum. For example, Garrison (2009) defines social presence as an ability that progresses along three stages (acquiring social identity, creating purposeful communication, and building relationships). Alternatively, the social presence model (SPM) by Whiteside and Garrett Dickers (2016) defines it as a critical literacy for cultivating emotion and relationships (Garrison, 2009; Whiteside and Garrett Dickers, 2016; Lowenthal and Snelson, 2017). Moreover, the available literature on social presence has persisted in exploring the concept through a one-dimensional lens, examining it mostly on the individual level when in fact social presence is a shared experience dynamically co-created by at least two individuals (Biocca and Harms, 2002). Although there are accounts addressing the theoretical intersubjective dimension of the construct, it seems that few (if any) have attempted to operationalize its measurement (Lowenthal and Snelson, 2017). Finally, the co-createdness of social presence also accentuates its continuous, dynamic, and fluctuating nature in the course of an interaction. Unfortunately, social presence's dynamic nature has seldom been conceptualized or measured in the context of online social interactions.

We therefore suggest adopting the biobehavioral synchrony model (Feldman, 2012) as a venue for assessing the dynamic and interpersonal nature of social presence during videoconferencing social interactions. A central mechanism underpinning human social communication in real life is the synchronization between interacting partners. Synchrony is a spontaneous and automatic interpersonal coordination which unfolds over the course of milliseconds in constant and contingent dynamic feedback loops. Synchrony is not merely a behavioral or psychological phenomenon but a multileveled biobehavioral system that contains the human capacity to coordinate behavioral, mental, and physiological processes between interactive partners during moments of social contact. The biological aspect includes the coordination of heart rhythms, hormonal activity, neural oscillations, and brain activations (Feldman, 2012, 2016, 2017). According to the biobehavioral synchrony model, physiological coordination is triggered in a bottom-up way and depends on the coordination of social action, such as motor activity, facial mimicking, or the synchrony of non-verbal interactive signals, including shared gaze, joint laughter, or mutual expression of positive affect. The developmental origins of the model maintain that interpersonal biobehavioral synchrony is a key feature of the mother–infant bond in mammals, where the mature maternal psycho-behavioral social neural network externally regulates the infant's immature neural network, helping it adapt and tuning it to social living. These early attachment experiences are then transferred in complex ways to other social affiliations throughout life, such as romantic relationships or close friendships (Levy et al., 2017). Measuring biobehavioral synchrony during moments of online social contact may provide the missing link in the field of remote social encounters: the accurate and appropriate measurement of social presence as it unfolds. The incorporation of the simultaneous measurement of physiological measures with behavioral observations of coordination in both interacting partners in the assessment of mediated interactions can enable the calculation of continuous interpersonal variables of interactive synchrony. Possible examples of physiological measures include heart rate variability (HRV) and galvanic skin response (GSR). These might provide insight into how regulated or stressed a communicator's autonomic nervous system is over the course of the interaction, while examples of simultaneous behavioral observations could be facial mimicking or joint expression of affect.

An examination of such behavioral and physiological variables will also allow us the opportunity to closely document and monitor the possible peculiar or even adverse psycho-physiological effects brought about by the flaws that typify the available technologies, which might hamper the desirable experience of social presence. In the midst of the pandemic, journalists and scientists have begun to conceptualize a phenomenon called “Zoom fatigue” to describe feelings of tiredness, anxiety, or worry resulting from overuse of virtual video communication platforms (Wienderhold, 2020). Empirical research into the underlying causes is still in its incipient stages, yet Bailenson (2021) has suggested a few possible theoretical explanations for this phenomenon, mostly centering on the psychological and physiological stress caused by the various design features that differentiate video platform-mediated interactions from real life face-to-face ones. Specifically, mirror anxiety may be triggered by the self-view feature of the platform, or one may feel physically trapped because of the need to stay within the camera's view. Third, there is the experience of “hyper-gaze,” or the perceptual experience of constantly having other people's gaze in one's field. Finally, there is the potential for cognitive overload, caused by intentional and effortful production and interpretation of non-verbal behavior and cues, occurring more naturally and subconsciously in real life face-to-face interactions (Bailenson, 2021; Fauville et al., 2021). The proposed interpersonal close monitoring of behavior and physiological arousal during the course of an interaction would be ideal for measuring the actual stress experienced by each participant and the way it might interrupt or facilitate one's ability to synchronize with one's communicating partner over the platform. Such findings might serve to guide the developers of these technologies in tailoring their designs to the characteristics and functions of our psycho-behavioral social communication brain network.

Over the past decades, there has been an exponential boom in neuroscientific studies of interpersonal social interaction (Schilbach et al., 2013; Redcay and Schilbach, 2019; Shamay-Tsoory and Mendelsohn, 2019). Moreover, recent technological developments in portable devices allow for the design of quick and lifelike experimental designs that would enable a paradigm shift toward monitoring the naturalistic real-life dynamics of social interactions. As such, this technology would be ideal for incorporating in the real-life monitoring of digital interactions.

To conclude, we suggest that the most comprehensive and up-to-date understanding of the way our nervous system interacts with our social behavior system in specific communication technologies should closely guide the design of telecommunication platforms. Indeed, the incorporation of an interpersonal perspective guided by the theoretical framework of the biobehavioral synchrony model in the study and measurement of social presence will pave the way for the crystallization of such an understanding. The advancement of technological innovations will forever proceed from the incremental adjustments that our biological system is able to make over time. Failing to “stay ahead of the ball” would cement humanity in a constant state of simply reacting to the challenges and disruptions technology brings upon human communication instead of proactively and skillfully orchestrating its development to serve our needs.

## Author Contributions

All authors have contributed to this manuscript's conceptualization and writing, contributed to the article, and approved the submitted version.

## Funding

This study was supported by a grant from the Israel Science Foundation (ISF-494/21). This study was partially based on the JG's dissertation studies carried out at Bar-Ilan University, Ramat-Gan, Israel.

## Conflict of Interest

The authors declare that the research was conducted in the absence of any commercial or financial relationships that could be construed as a potential conflict of interest.

## Publisher's Note

All claims expressed in this article are solely those of the authors and do not necessarily represent those of their affiliated organizations, or those of the publisher, the editors and the reviewers. Any product that may be evaluated in this article, or claim that may be made by its manufacturer, is not guaranteed or endorsed by the publisher.
